# The Monarch Butterfly as a Model for Understanding the Role of Environmental Sensory Cues in Long-Distance Migratory Phenomena

**DOI:** 10.3389/fnbeh.2020.600737

**Published:** 2020-12-03

**Authors:** Patrick A. Guerra

**Affiliations:** Department of Biological Sciences, College of Arts and Sciences, University of Cincinnati, Cincinnati, OH, United States

**Keywords:** monarch butterfly, animal migration, migratory syndrome, sensory cue degradation, sensory pollution

## Abstract

The awe-inspiring annual migration of monarch butterflies (*Danaus plexippus*) is an iconic example of long-distance migratory phenomena in which environmental sensory cues help drive successful migration. In this mini-review article, I begin by describing how studies on monarch migration can provide us with generalizable information on how sensory cues can mediate key aspects of animal movement. I describe how environmental sensory cues can trigger the development and progression of the monarch migration, as well as inform sensory-based movement mechanisms in order to travel to and reach their goal destination, despite monarchs being on their maiden voyage. I also describe how sensory cues can trigger season-appropriate changes in migratory direction during the annual cycle. I conclude this mini-review article by discussing how contemporary environmental challenges threaten the persistence of the monarch migration. Environmental challenges such as climate change and shifting land use can significantly alter the sensory environments that monarchs migrate through, as well as degrade or eliminate the sources of sensory cues that are necessary for successful migration.

## Introduction

### Sensory Ecology of Long-Distance Animal Migration

In many animal species, individuals can exhibit locomotory behavior and movement patterns across varying temporal (e.g., from seconds to years) and spatial (e.g., from local natal patches to round-the-world journeys) scales. For many of these phenomena, the movement of individuals is goal-driven, such that individuals are moving to travel to specific locations that contain key resources that are often necessary for survival or that can promote individual fitness. Long-distance migration is an example of goal-oriented animal movement phenomena that typically occurs seasonally, with individuals undergoing journeys that can span thousands of miles. Migration can be an adaptive strategy, as individuals travel to take advantage of seasonally available resources found at different locations, such as specific plant hosts, shelters, feeding areas, or breeding grounds. Also, individuals can migrate to escape predictably deteriorating habitats for locations with more hospitable environmental conditions, and then return to their original habitats once conditions have improved or have returned to normal (Dingle, 2014).

Environmental sensory cues can strongly mediate and modulate the goal-directed migratory movement of individuals. For instance, sensory cues that occur with specific timing and that are correlated with the arrival of deteriorating conditions can trigger the development of phenotypic traits in individuals that facilitate migratory movement, as well as initiate the onset of migration. To travel to and reach their destination during migration, individuals will often use or must rely on sensory cues that they also derive from their environment. These sensory cues can vary in both their form and function. For example, individuals might rely on a single cue that can reliably direct their movement towards their goal when still very far away. Once near their destination, individuals might then use sensory cues as guideposts that trigger other behaviors or sensory processes for finding their goal. These sensory cues might also serve as beacons of the destination itself, thereby allowing migrants to recognize, localize, and stop at their goal (Reppert et al., 2010; Mouritsen, 2018). Finally, sensory cues can inform individuals as to if and when they can remigrate back.

### Monarch Butterfly Long-Distance Migration

The annual multigenerational migratory cycle of the monarch butterfly (*Danaus plexippus*) is an iconic example of long-distance animal movement phenomena. Found in many different parts of the world, perhaps the most famous population of this species consists of the butterflies that live east of the Rocky Mountains in North America. Each fall, millions of monarchs in Eastern North America leave their summer breeding grounds in Southern Canada and the Northern United States and fly southwards to migrate to their overwintering areas in Central Mexico. These overwintering sites consist of a handful of coniferous fir groves (oyamel) high atop the Transvolcanic Mountains in the state of Michoacán upon which butterflies will aggregate and roost during the winter (Urquhart, 1987). Upon the arrival of spring, these same monarchs leave the overwintering sites, flying northwards to return and start repopulating the southern tier of the United States. The offspring of these spring remigrants, i.e., spring populations of butterflies, continue the migratory cycle by flying northwards. The migratory cycle ends with a summer generation of non-migratory butterflies that repopulates the most northern regions of the monarch habitat range. This migratory cycle begins anew when the next generation of monarchs flies southwards in the fall (Reppert et al., 2016). A similar, albeit smaller scale fall migration occurs with the population of monarchs that live west of the Rocky Mountains. Monarchs of the Pacific Northwest and Northern California fly southwards to overwintering sites along the Pacific Coast in California. In contrast to the overwintering fir groves in Mexico, Western monarchs overwinter on evergreen Monterey Pine and Eucalyptus trees (Reppert and de Roode, 2018). In the spring, remigrants leave the overwintering sites, and successive generations fly northwards to repopulate the habitat range. Fall monarchs from the Southwestern United States also migrate, with monarchs reaching overwintering sites in either California or Mexico (Morris et al., 2015). Outside of North America, monarchs in Eastern Australia can also migrate to seasonally appropriate habitats, in the same manner as their counterparts in the Northern Hemisphere (James and James, 2019; Nail et al., 2019). This group of fall migrants will roost on trees (e.g., native prickly paperbark) that are different from those used by monarchs in either Eastern or Western North America (James, 1993; Nail et al., 2019).

In contrast to these regions with populations of monarchs that display directional flight and migrate, other monarchs can be found in several areas around the world in which they are considered non-migratory, e.g., Florida, Hawaii, and New Zealand. These monarchs can be found as year-round residents or will engage in winter breeding (Reppert and de Roode, 2018; Nail et al., 2019). Although monarchs from these populations have been observed to fly only short distances relative to conspecifics that migrate (e.g., monarchs in New Zealand; Wise, 1980), it remains unknown if these individuals also display oriented flight, especially flight in the seasonally appropriate direction, the hallmark trait of migratory monarchs. It is possible that monarchs from these populations display directional flight, but the distances of their flights are simply limited by geographical constraints, e.g., living on a relatively small island in the middle of the ocean. Although it is possible that traits associated with migration, e.g., oriented flight behavior, can be quickly selected out to produce populations of migratory species that are non-migratory, such traits might remain in the population due to evolutionary inertia (Alerstam, 2006) or exist despite large differences in the movement ecology of populations (Scanlan et al., 2018). For instance, translocated nonanadromous Atlantic salmonids with no recent history of migration, can display similar directed responses to local orientation cues as native Pacific salmonids (Scanlan et al., 2018). Monarchs from populations now considered non-migratory might retain and still be capable of using orientation mechanisms like migratory conspecifics in a similar manner. Behavioral studies assaying the flight orientation of putative non-migratory monarchs at these locations, e.g., flight simulator trials (Mouritsen and Frost, 2002), can address this.

### Role of Environmental Sensory Cues in Monarch Butterfly Migration

**Figure 1:**
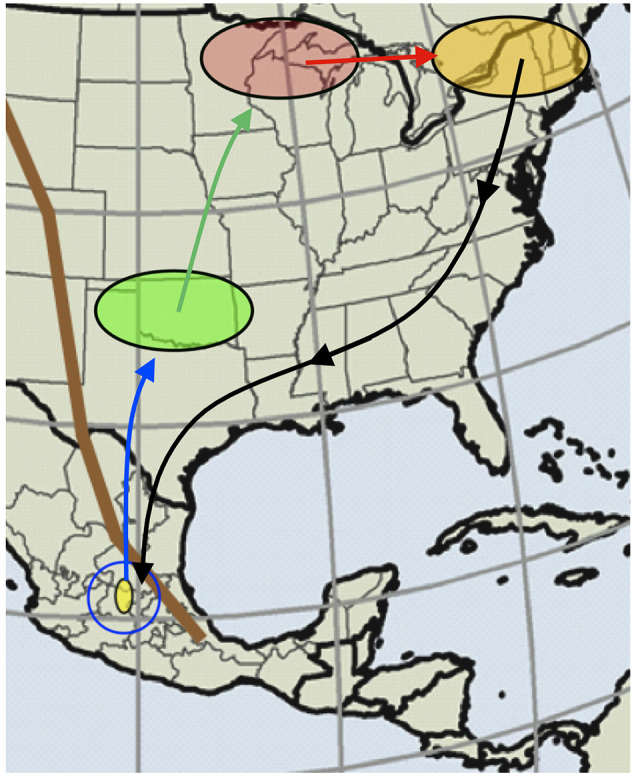
Monarch butterflies use sensory cues to facilitate their annual multigenerational migratory cycle. Shown is the Eastern North American population of butterflies that live east of the Rocky Mountains (brown line). In the late summer and early fall, developing monarchs in the upper regions of the monarch habitat range sense environmental cues that induce the monarch migratory syndrome and that initiate the southwards fall migration (orange oval). These fall migrants use various sensory-based compass mechanisms to guide them southwards during their migratory journey (black line) and potentially use cues once close to their destination (blue circle) that allow them to locate and stop at the overwintering sites in Central Mexico (yellow oval). After receiving a cold trigger while overwintering that recalibrates their compass mechanisms for the return journey, these monarchs remigrate northwards during the spring (blue line). Spring monarchs (green oval), the offspring of spring remigrants, continue the migratory cycle by traveling northwards (green line). These spring monarchs potentially use the same navigational mechanisms as fall conspecifics, but that are calibrated by sensory cues during development for northwards flight instead (green oval). Successive generations of monarchs fly northwards until they repopulate the northern breeding grounds of the monarch range (red oval). The migratory cycle ends, once monarchs experience cues that either signal them to stop or that do not trigger oriented flight behavior (red oval). Summer butterflies repopulate the most northern areas of the monarch range (red oval), and once their offspring experience the necessary cues (orange oval), the migratory cycle begins anew. Figure modified from Guerra and Reppert (2015).

Research using the monarch as a model system has provided useful and generalizable information on animal migration at different mechanistic levels, from the behavioral, neural, molecular, and genetic substrates of this phenomenon (Reppert et al., 2016; Reppert and de Roode, 2018; Merlin et al., 2020). In particular, previous studies have demonstrated the key role of environmental sensory cues for successful migration, with sensory cues playing a vital function at almost all stages of the monarch migratory cycle (Guerra and Reppert, 2015; Figure 1).

Environmental sensory cues are necessary for monarch migration to occur, as the sensing of cues correlated with the arrival of fall, i.e., decreasing photoperiod and cooler and fluctuating temperatures (Goehring and Oberhauser, 2002; Freedman et al., 2018), can help induce the monarch migratory syndrome in individuals. In contrast to summer monarchs that are non-migratory, fly non-directionally, and are reproductively active (Zhu et al., 2009), monarchs that develop in the late summer and early fall can sense cues that induce the development of morphological (e.g., wings that are redder and have more melanization—Hanley et al., 2013; Satterfield and Davis, 2014; more elongated wings—Satterfield and Davis, 2014; larger forewings—Li et al., 2016), biochemical (e.g., lower juvenile hormone titers—Zhu et al., 2009), reproductive (i.e., diapause—Goehring and Oberhauser, 2002), and sensory traits (e.g., time-compensated sun compass use to maintain directional flight—Zhu et al., 2009) that can facilitate long-distance migration. The onset, timing, and pace of the migration appear to also be regulated by environmental sensory cues. For instance, the timing and pace of the fall migration in Eastern North America are associated with migratory monarchs sensing specific celestial cues (i.e., the sun’s position in the sky, specifically the sun angle at solar noon) and environmental parameters (temperature and daylength; Taylor et al., 2019).

Although on their maiden voyage, fall migrants are capable of traveling to their overwintering destinations by using various innate sensory-based orientation mechanisms to guide migratory flight (compass sense—Reppert et al., 2016). Eastern North American fall monarchs can use a time-compensated sun compass, the dominant orientation mechanism of migratory monarchs, to maintain proper southwards flight directionality (Perez et al., 1997; Mouritsen and Frost, 2002; Froy et al., 2003). Monarchs use the sun’s position in the daytime sky as a visual cue to maintain a southwards flight orientation. To correct for the apparent movement of the sun across the sky throughout the day, monarchs use timing information derived from antennal circadian clocks that are entrained to local photoperiodic conditions, to compensate for the sun’s movement (Merlin et al., 2009; Guerra et al., 2012). Interestingly, recent work has shown that even non-migratory monarchs can use such sun visual cues for orientation during flight (Franzke et al., 2020). On overcast days, a time when the sun’s position is occluded, Eastern North American fall migrants can use a magnetic compass as a backup for maintaining southwards directionality. In contrast to the more familiar magnetic compass that distinguishes North from South by measuring the polarity of geomagnetic field lines to compare North vs. South (a “polarity compass”), the monarch magnetic compass utilizes the inclination angle of the geomagnetic field as a cue for directionality (an “inclination compass”). Here, monarchs can sense how geomagnetic field lines intersect the Earth’s surface, with field lines ranging from parallel to the Earth’s surface at the equator (0° inclination angle), to field lines intersecting the Earth’s surface perpendicularly at either pole (90° inclination angle). As the inclination angle of the geomagnetic field predictably covaries with latitude, fall migrants can determine if they are flying either equatorward or polewards (Guerra et al., 2014). In addition to using inclination angle cues for directionality, this can allow migratory animals with a magnetic sense to use these cues as part of a geomagnetic coordinate system that can provide positional or map information during migration (Mouritsen, 2018). To detect magnetic fields, monarchs require exposure to ultraviolet A/B light wavelengths, with the putative magnetosensors located in the antennae (Guerra et al., 2014). Though the flight directionality and compass use of fall migrants in Western North America and Australia have yet to be directly tested, it is highly probable that butterflies in these regions also use the same sensory cues for flight directionality during migration, and employ these compass mechanisms with the same morphological substrates (Merlin et al., 2009; Heinze and Reppert, 2012), neural circuitry (Guerra et al., 2012; Heinze et al., 2013; Shlizerman et al., 2016), and genetic architecture (Zhan et al., 2014).

Compass senses only provide directional information and do not allow monarchs to know where they are relative to their goal. As each generation of fall monarchs is naïve to the location of the overwintering sites, monarchs must possess innate mechanisms that allow them to find and stop at these locations. It remains a great mystery of how monarchs achieve this goal-oriented task each year. A possible mechanism is *via* a map sense that can provide positional information, with one type involving monarchs using their magnetic sense (Guerra et al., 2014) for identifying the specific geomagnetic signatures of the overwintering sites. The recognition and localization of the overwintering grounds by sensing magnetic cues correlated with these locations (a type of beacon cue) can assist monarchs with finding the appropriate groves of trees upon which they aggregate and overwinter (Mouritsen, 2018). Alternatively, monarchs might instead use beacon cues that indicate the overwintering sites independent of a map sense. Like the use of signals by insects for attracting individuals from far away to form large groups (e.g., aggregate male calling song—Guerra and Mason, 2005; aggregation pheromone—Allison and Cardé, 2016), monarchs might use cues emanating from the overwintering sites, e.g., olfactory cues given off by the trees (Reppert and de Roode, 2018), to form their massive overwintering aggregations. Stopping at the overwintering sites might also be a form of habitat selection, in which monarchs are searching for suitable microclimates for overwintering. For example, the microclimate of monarch overwintering sites differs from that outside the tree groves and provides temperatures that are low enough to keep metabolic costs low for overwintering but are not so cold that they lead to freezing and death (Urquhart and Urquhart, 1976). Monarchs might therefore stop at the overwintering sites, by using temperature as an environmental cue once close. It is also possible that monarchs stop at the general area of the overwintering sites since they simply no longer perceive a specific sensory cue that signals to continue migratory flight, such as the sun’s angle at solar noon, i.e., loss of cue hypothesis (Taylor et al., 2019). Here, monarchs might then home in on beacon cues for locating the overwintering sites. The cues used to stop at their respective overwintering sites by the different migratory populations might be different and reflect local adaptation, as the geographic locations and trees used for aggregation differ between the groups (see above). In contrast, migrants regardless of region might utilize a common mechanism for stopping based on their shared search for appropriate microclimates for overwintering. Indeed, this might be the case for at least North American monarchs, in which overwintering temperature conditions are similar for both Eastern and Western migrants at the overwintering sites (Guerra and Reppert, 2013).

Finally, sensory cues are also important for the completion of the migratory cycle. Shown with Eastern North American monarchs, migrants need to be exposed to cold temperatures as experienced during overwintering in Mexico, to fly with the appropriate return flight directionality (*via* a recalibrated time-compensated sun compass) for remigration during the spring. Without exposure to such temperatures, monarchs continue to fly with fall flight directionality (Guerra and Reppert, 2013), which can prevent them from remigrating properly. Although still unknown, spring monarchs might use identical compass mechanisms, but with reversed directionality relative to fall monarchs for remigration. Similar to fall monarchs, the remigration directionality of spring monarchs might be induced by sensory cues associated with the season. These cues, however, should display a pattern in spring that is shifted 180° from that in late summer and early fall, such as increasing daylength and warming temperatures. The termination of the migratory cycle with the accompanying loss of directional flight observed in monarchs might also be due to the sensing of environmental cues, e.g., the decrease in the rate of change of increasing daylength that culminates with the summer solstice (Taylor, 2013). Moreover, the longer daylengths and higher temperatures of late spring and summer do not produce butterflies with the migratory syndrome.

## Discussion

Although our knowledge on the fundamental role of environmental sensory cues on monarch migration has increased over the past few decades, information on how contemporary changes in the sensory environment of monarchs might affect the migratory cycle remains lacking. This gap in our knowledge on this particular risk to sensory cue usage represents a potential danger to monarchs.

### The Effects of Urbanization Threaten Monarch Migration

Major threats to the sensory environment of monarchs are those brought about by human activity (Kelley et al., 2018), such as shifting land usage related to urbanization. For example, human-induced highway noise as experienced by monarch larvae at roadside habitats can be a source of physiological stress (Davis et al., 2018). It is unknown how such physiological stress might affect the development, health, and survivorship of individuals, in particular individuals that will develop into migrants or adult migrants already en route. Urbanization is also a significant source of nighttime light pollution (NLP), such that urban areas with significant levels of NLP can present monarchs that develop and live there, or are just passing through while migrating, with dramatically altered daily light levels and photoperiods (Gaston et al., 2014). As environmental light cues with the appropriate characteristics and proper circadian clock function are important for proper monarch migration, the NLP of urban areas along the migratory routes of monarchs might significantly disrupt the entire migratory cycle. In urban areas, NLP might artificially prolong the subjective daytime hours of monarchs as observed for individuals of other migratory species (Dominoni and Partecke, 2015) or produce constant light conditions with properties (e.g., significant intensity and relevant wavelengths of light) that can significantly disrupt normal circadian clock function. NLP in urbanized areas might significantly alter or obliterate the cue of decreasing photoperiods that induce the migratory syndrome (Goehring and Oberhauser, 2002) and instead lead to the production of non-migratory individuals even during the fall. Constant light conditions due to NLP can disrupt the development of monarchs, e.g., eclosion behavior (Froy et al., 2003), as well as perturb the antennal circadian clock function of adult migrants, preventing correct flight orientation during migration (Merlin et al., 2009). As many initiatives to conserve the monarch are conducted in urban areas (Baker and Potter, 2019), research on how urban NLP affects monarch migration is now needed to prevent or mitigate any unintended consequences of current and future conservation efforts. Fortunately, it is possible to reduce the negative ecological effects of NLP in urban areas, by using better lighting technologies and altering human behavior and lighting strategies at night (Gaston et al., 2012). Urbanization can also lead to human-induced electromagnetic noise, which can disrupt magnetic compass orientation in migratory animals (Engels et al., 2014). As monarchs can also use a magnetic compass for orientation during migration (Guerra et al., 2014), noise in this sensory modality is another type of sensory pollution that can prevent successful migration.

### The Loss of Important Sensory Cues

Habitat loss and degradation are also areas of vulnerability for the persistence of the monarch migration. Central to the monarch migratory cycle are the overwintering sites that provide monarchs with a suitable microclimatic overwintering refuge. As migrating monarchs might use beacon cues provided by the overwintering grounds for locating these sites, the current deforestation and degradation of these areas, such as at the overwintering sites in Mexico of Eastern North American migrants (Vidal et al., 2013; Malcolm, 2018), might significantly reduce the strength, quality, or occurrence of cues emanating from these areas that guide monarchs. Also, as the thermal microclimate of the overwintering sites produces a “cold trigger” cue critical for recalibrating the flight directionality of migrants for proper remigration during the spring (Guerra and Reppert, 2013), worldwide challenges such as global warming and overall global climate change might currently attenuate this coldness cue and imperil the future persistence of this critical sensory cue at the overwintering sites. Without this coldness cue, it is possible that the migratory cycle can be broken, since monarchs may not return to the breeding areas of their habitat range. Unfortunately, previous modeling of the persistence of overwintering sites for migratory monarchs, such as the Monarch Butterfly Biosphere Reserve in Central Mexico, suggests that suitable overwintering habitat for monarchs at current sites might completely disappear by the end of this century (Sáenz-Romero et al., 2012). Therefore, research on the sensory ecology of monarch migration should continue to focus on identifying how monarchs locate the overwintering sites and what cues are used to do so. Once these cues are identified, it might be possible to identify, monitor, and protect new locations that provide these same cues and that monarchs find suitable for overwintering. Similarly, artificial and better-protected overwintering areas could be constructed to attract migrating monarchs. As done with other long-distance migratory animals (e.g., studies delineating the sensory-based orientation and navigational mechanisms of marine species such as sea turtles and salmonid fishes; Putman, 2018), by further studying and understanding the sensory capabilities of monarchs and the cues that they use for migration, we will be better equipped to save this wonder of nature, as well as other animal movement phenomena that face similar challenges.

## Author Contributions

The author confirms being the sole contributor of this work and has approved it for publication.

## Conflict of Interest

The author declares that the research was conducted in the absence of any commercial or financial relationships that could be construed as a potential conflict of interest.
